# HBV is a risk factor for poor patient prognosis after curative resection of hepatocellular carcinoma: A retrospective case–control study

**DOI:** 10.1097/MD.0000000000004224

**Published:** 2016-08-07

**Authors:** Zhonghu Li, Xin Zhao, Peng Jiang, Senlin Xiao, Guo Wu, Kai Chen, Xi Zhang, Hui Liu, Xiuguo Han, Shuguang Wang, Xiaowu Li

**Affiliations:** Hepatobiliary Surgery Institute, Southwest Hospital, Third Military Medical University, China.

**Keywords:** hepatitis B virus, hepatocellular carcinoma, recurrence, survival

## Abstract

Controversy exists regarding pathological factors affecting the prognosis of hepatocellular carcinoma (HCC) patients with hepatitis B virus (HBV-HCC). Their postoperative clinical behaviors and the exact HBV Deoxyribonucleic Acid (DNA) thresholds that distinguish good and poor prognoses are unknown. This study aimed to compare clinicopathological, pre- and postoperative clinical factors and overall and recurrence-free survival (RFS) between HBV-HCC patients and nonhepatitis B and nonhepatitis C HCC (NBC-HCC) patients to determine the optimal prognostic HBV DNA threshold.

Data from 1440 patients with HBV-HCC and NBC-HCC who underwent curative hepatectomy were retrospectively analyzed.

Liver function in the HBV-HCC group was significantly worse than in the NBC-HCC group. Compared with NBC-HCC patients, HBV-HCC patients had significantly more vascular invasion and advanced HCC. The HBV-HCC patients also had significantly worse liver function and more complications. Further survival analysis showed significantly lower overall and RFS rates and a higher early recurrence rate in the HBV-HCC group. Univariate analysis indicated that HBV was a risk factor for overall and RFS. Finally, X-tile analysis revealed that the optimal HBV DNA cutoff points for predicting RFS and overall survival in HCC patients were 10,100 and 12,800 IU/mL, respectively.

After hepatectomy for HCC, HBV-HCC patients had more complications and a worse prognosis than NBC-HCC patients. Antiviral therapy should be considered before hepatectomy in patients with high (more than approximately 10^4^ IU/mL) HBV DNA levels.

## Abstract 2

Supplemental Digital Content is available in the text

## 1 Introduction

Hepatocellular carcinoma (HCC) is the sixth most common cancer in the world and the second leading cause of cancer-related death.^[1]^ Among the many etiological risk factors that are associated with HCC, the most important is chronic hepatitis B virus (HBV) infection, as approximately 30% to 40% of HBV carriers eventually develop HCC.^[2,3]^ Therefore, HBV-related HCC (HBV-HCC) is a considerable worldwide problem for human health, especially in East Asia.^[4]^

By far, the most important strategy for HCC management is early diagnosis and radical treatment.^[5]^ Hepatic resection is still the main treatment.^[6,7]^ With the recent advances in medicine and technique, overall posthepatectomy survival rate of HCC patients has increased in recent years.^[8]^ However, an increasing number of studies have shown that postoperative tumor recurrence in the remnant liver is common and can be fatal.^[9]^ Thus, identifying predictive factors for HCC recurrence is extremely important.^[10]^ Some studies have reported that HCC patients without HBV have a poorer prognosis than their counterparts due to delays in diagnosis and the presence of more advanced-stage disease.^[11,12]^ However, other studies have shown that HBV may worsen liver function, leading to complications including hepatocarcinogenesis, thereby affecting recurrence and survival rates in HCC.^[13–15]^

Unfortunately, only a few studies have compared the clinicopathological characteristics and prognosis after hepatectomy among patients with and without HBV infection, and their results have conflicted.^[13,16,17]^ Moreover, some studies have shown that HBV Deoxyribonucleic Acid (DNA) levels may be associated with HCC recurrence, but each study grouped HBV DNA levels (high or low) differently.^[5,18–20]^ In addition, some studies examined an insufficient number of patients, and others considered different demographic characteristics. These differences may explain the inconsistent results. Recently, many studies have shown that preoperative anti-HBV therapy can improve the prognosis of HCC. However, whether the HBV DNA should be decreased to a normal level or to some other threshold before surgery is unknown.^[14,21,22]^

This study aimed to compare the clinicopathological characteristics and prognosis after hepatic resection between patients with and without HBV and to identify an optimal HBV DNA value to distinguish good and poor prognoses. Therefore, we retrospectively analyzed the clinical data from 1440 resected HCC patients at our institute.

## 2 Patients and methods

### 2.1 Study patients

Between January 2008 and December 2012, a retrospective study was conducted on HCC patients who underwent partial hepatectomy at the Hepatobiliary Surgery Institute, Southwest Hospital, Third Military Medical University, China. The exclusion criteria included the following: extrahepatic metastasis, Child-Pugh class C disease, lack of hepatitis B surface antigen (HBsAg) status data, and noncurative hepatectomy, other viral hepatitis (or mixed types) have also been excluded in this study. The 1440 HCC patients included in the study were classified according to hepatitis virus infection (i.e., HBsAg) status into an HBV-HCC group (n = 1200, 83.3%) and an NBC-HCC group (n = 240, 16.7%). The serum HBV and HCV DNA of each patient were tested by the polymerase chain reaction (PCR) assay (ABI 7300; Applied Biosystems, Foster City, CA), the linear range of quantification is from 200 to 2 × 106 IU/mL.

All patients underwent chest radiography, ultrasonography, and contrast-enhanced computerized tomography (CT) or magnetic resonance imaging (MRI). Laboratory testing included HBsAg, hepatitis C virus antibody, α-fetoprotein (AFP), alanine aminotransferase (ALT), aspartate aminotransferase (AST), albumin (ALB), total bilirubin (T-bil), indocyanine green 15′ retention test (ICG-R15), prothrombin time (PT), and blood platelet count. HBV DNA was analyzed using quantitative real-time PCR (q-PCR). The presence of cirrhosis and the nature and size of the tumor were all confirmed by final pathological examination. The indication for each hepatectomy was fully assessed by a departmental tumor board. Intraoperative ultrasonography was always used to detect invisible, nonpalpable nodules. The tumor node metastasis (TNM) malignant tumor classification system of the Union for International Cancer Control was used to classify the HCC stage, this study was approved by the Ethics Committee of Southwest Hospital, and all patients provided written informed consent.

### 2.2 Clinical outcome assessment

After discharge, the patients were prospectively followed up using AFP levels, contrast-enhanced ultrasound, enhanced CT, or MRI at 3-month intervals for the first year and then at gradually increasing intervals. The final prognosis was followed by the Clinical Follow-up Center of our department at 3-month (at most) intervals until death. Based on the time of recurrence from the date of hepatectomy, recurrences were classified as early (within 2 years) or as late (more than 2 years).

### 2.3 Selection of the HBV DNA cutoff point

We used X-tile 3.61 (Yale University, New Haven, CT) software to determine the optimal cutoff point for the exact levels of HBV DNA that predicted overall survival (OS) and recurrence-free survival (RFS) in HCC patients. The X-tile program split the HBV DNA cohort randomly into a matched training and validation set to select optimal cutoff points. Selections were based on a log-rank χ^2^ statistic for every possible division of the cohort expression data, which were then divided into either 2 or 3 optimal groups based on the continuous input data. A two-dimensional graph with its corresponding survival curves was plotted, where each colored pixel was proportional to its χ^2^ value. The program automatically calculated the maximum χ^2^ value, which served as a cutoff point to identify the level of HBV DNA that predicted prognosis.

### 2.4 Statistical analysis

Categorical clinical variables of the HBV-HCC and NBC-HCC groups were compared by the χ^2^ test or the Fisher exact test, continuous variables were compared using the Kruskal–Wallis test. Patient OS and RFS rates after surgical resection were calculated using the Kaplan–Meier method. The risk factors of OS and RFS after hepatectomy were evaluated by the univariate and the multivariate Cox proportional hazards models. The variables of the multivariate analysis were determined if their *P* values were less than 0.05 during the univariate analysis. The forward left-to-right, rightmost derivation method was adopted during the multivariate analysis to avoid the multicollinearity. The *P* value for a two-tailed test of less than 0.05 was considered statistically significant. All statistical analyses were performed using SPSS 19.0 for Windows (IBM, Chicago, IL).

## 3 Results

### 3.1 Clinical baseline characteristics of the study participants

Baseline clinical characteristics of the 2 patient groups (HBV and NBC) are summarized in Table 1. Compared with the NBC-HCC patients, the HBV-HCC patients were younger, with a higher proportion of males. In particular, the rate of comorbidities was significantly higher in the NBC-HCC group than in the HBV group. HBV-HCC patients had significantly higher levels of ALT, AST, T-bil, and PT. In addition, HBV-HCC patients were significantly more likely to have liver cirrhosis and Child class B disease, along with significantly lower serum ALB levels and platelet counts. HBV-HCC patients had significantly higher AFP levels and more advanced HCC based on the TNM stage and the vascular invasion ratio. However, we did not find statistically significant differences in tumor size, tumor number, or peripheral invasion ratio.

**Table 1:**
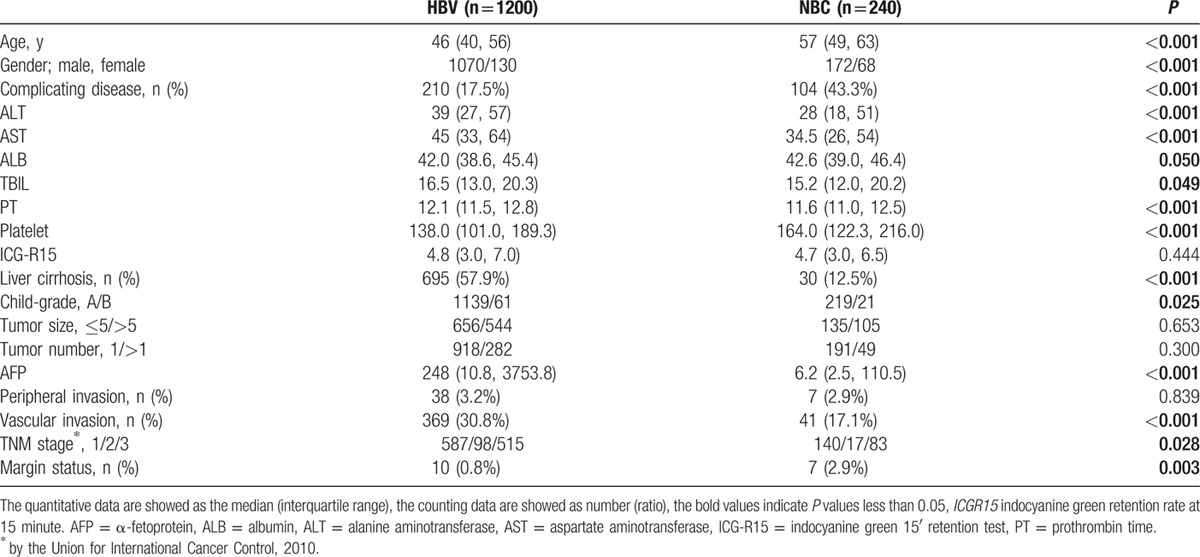
Clinical characteristics in the 1440 patients with hepatocellular carcinoma who underwent hepatectomy.

### 3.2 HBV-HCC patients had worse postoperative liver function and complications

Surgical data from all HCC patients were also investigated, but no significant differences were found in hepatic segmentectomy, hilar clamping, blood loss, or blood transfusion between the HBV- and NBC-HCC groups. However, we found that NBC-HCC patients were more likely to need additional surgery than HBV-HCC patients (Table 2). Further detailed study demonstrated that the NBC-HCC patients had a higher biliary surgery ratio, while the differences in splenectomy, portal venous thrombectomy, and diaphragmatic resection were not significant (Table S1).

**Table 2:**
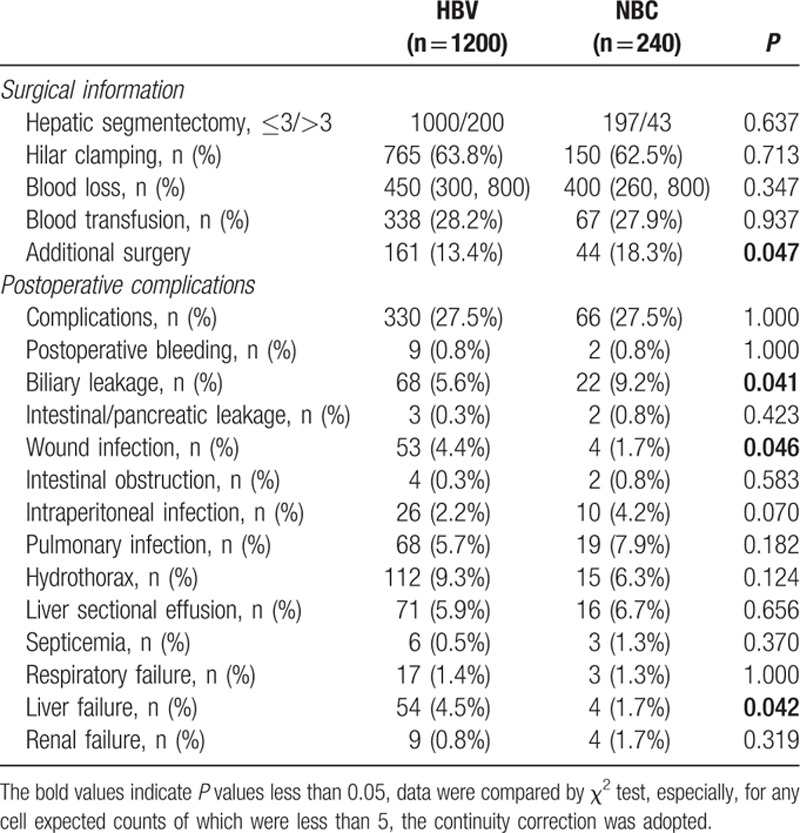
Surgical and postoperative complication information in the 1440 patients with hepatocellular carcinoma who underwent hepatectomy.

**Figure 1:**
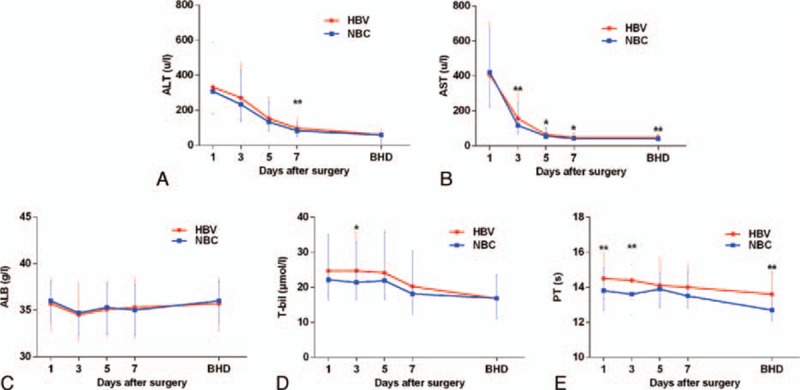
Comparisons of liver functions after hepatectomy between hepatocellular carcinoma patients with hepatitis B virus and nonhepatitis B and nonhepatitis C hepatocellular carcinoma patients. The levels of alanine aminotransferase (A), aspartate aminotransferase (B), albumin (C), total bilirubin (D), and prothrombin time (E) were analyzed on days 1, 3, 5, and 7 after surgery and before hospital discharge. All comparisons were made using the Mann–Whitney *U* test, the statistical results were showed by median (interquartile range).

To further compare postoperative liver function in the HBV and NBC patients, we collected detailed data from every patient for ALT, AST, ALB, T-bil, and PT on postoperative days (POD) 1, 3, 5, and 7 and before hospital discharge (BHD). Our results showed that, compared with NBC-HCC patients, the ALT levels of HBV-HCC patients were significantly higher on POD 7; their AST levels were significantly higher on POD 3, 5, 7, and BHD. The T-bil levels of HBV-HCC patients were significantly higher on POD 3; their PT was also significantly higher on POD 1 and 3 and BHD. In addition, we did not find any significant differences in the ALB levels of HBV-HCC patients due to additional interventions. It was clear that the postoperative liver functions of HBV-HCC patients recovered more slowly than the NBC-HCC patients (Fig. 1, Table S2).

We also observed 14 types of postoperative complications. Although HBV-HCC patients had a similar complication rate, HBV-HCC patients showed higher wound infection and hepatic failure ratios but had lower biliary leakage rates. Rates of other complications were comparable between the 2 groups (Table 2).

### 3.3 HBV-HCC patients had worse OS and RFS

**Figure 2:**
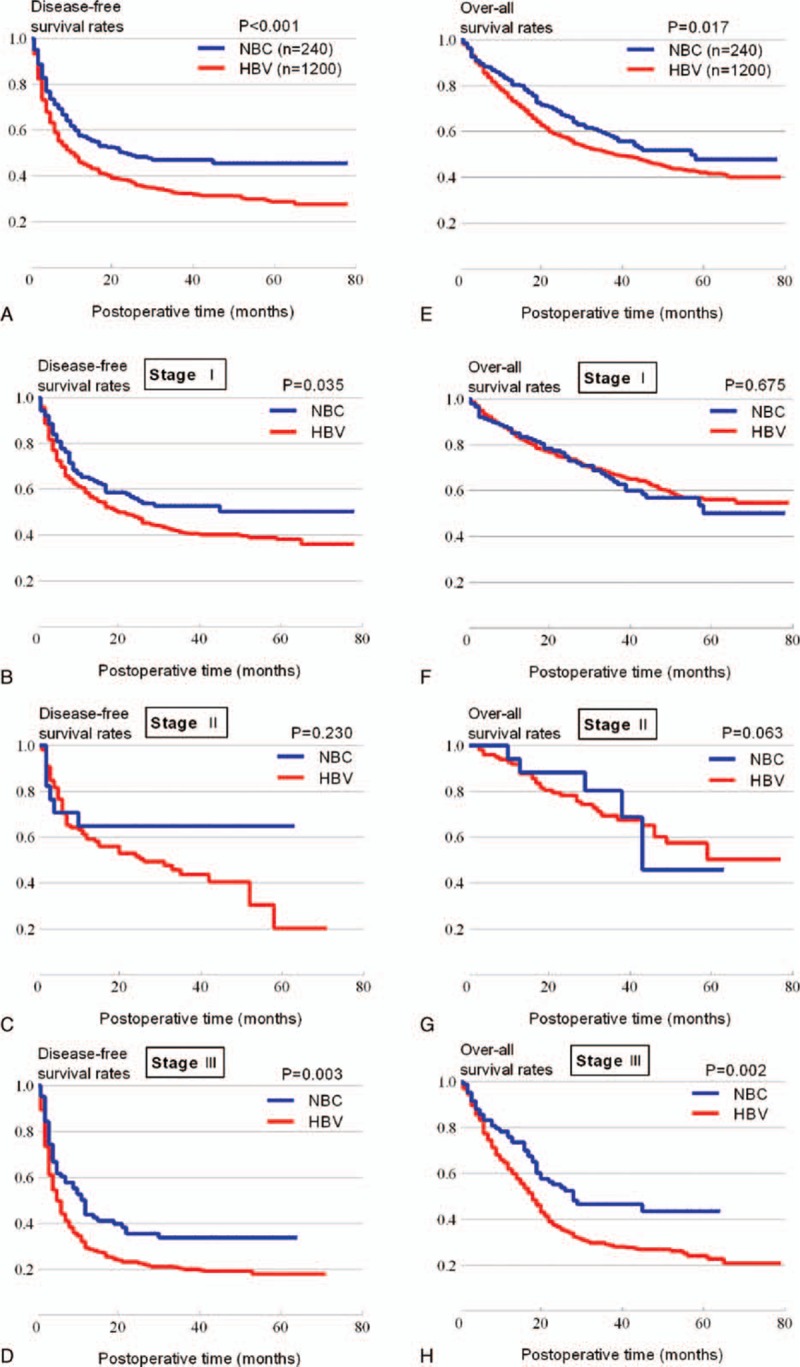
Comparisons of survival outcomes after hepatectomy between hepatocellular carcinoma patients with hepatitis B virus and nonhepatitis B and nonhepatitis C hepatocellular carcinoma patients. The recurrence-free survival (RFS) (A) and overall survival (OS) (E) rates were compared. The RFS rates were further stratified by TNM tumor stage into stage I (B), stage II (C), and stage III (D), while the OS rates were also stratified into stage I (F), stage II (G), and stage III (H), all comparisons were made using the log-rank test.

The median follow-up in the 1440 patients was 27 months (ranging from 1–85 months). Fig. 2 shows the prognosis after hepatic resection. The 1-, 3-, and 5-year OS rates in the HBV-HCC group were 74.8%, 50.8%, and 42.1% versus 82.4%, 58.8%, and 47.7% in the NBC-HCC group (*P* = 0.017), respectively. However, the difference in the RFS rate was greater than the OS rate, with lower median survival times and survival rates (Fig. 2A and E), The 1-, 2-, and 3-year RFS rates in the HBV-HCC group were 46.2%, 37.9%, and 32.5% versus 57.7%, 49.5%, and 47.0% in the NBC-HCC group, respectively (*P* < 0.001). Further, we found that the HBV-HCC group had a significantly higher early recurrence (within 2 years) rate (Table S3). Therefore, this study mainly used RFS rate comparisons between the 2 groups. Similar comparisons of the OS are reported in the supplemental data.

When the patients were stratified according to the TNM staging system, no significant differences in RFS were observed between the 2 groups in patients with TNM stage II tumors, HBV-HCC patients had significantly worse RFS in stages I and III compared with NBC-HCC patients (Fig. 2B–D). Similar results were found for OS in TNM stage II and III (Fig. 2F–H).

### 3.4 HBV is an independent risk factor for RFS after hepatic resection

Fourteen clinicopathological variables were screened as risk factors for HCC recurrence using univariate analysis. We found that age, sex, ICG-R15, and tumor number were not prognostic factors. The remaining factors, including HBV, TNM stage, and vascular invasion, were risk factors for RFS in patients with HCC (Table 3). Further, multivariate analysis showed that only HBV, AFP, tumor size, vascular invasion, and additional surgery were independent risk factors for RFS. HBV-HCC patients had a significantly worse RFS rate than NBC-HCC patients (hazard ratio = 1.274; 95% confidence interval: 1.034–1.569). Similar results were found in univariate and multivariate analyses for OS. HBV was also a risk factor (although nonindependent) for OS in HCC patients (Table 4).

**Table 3:**
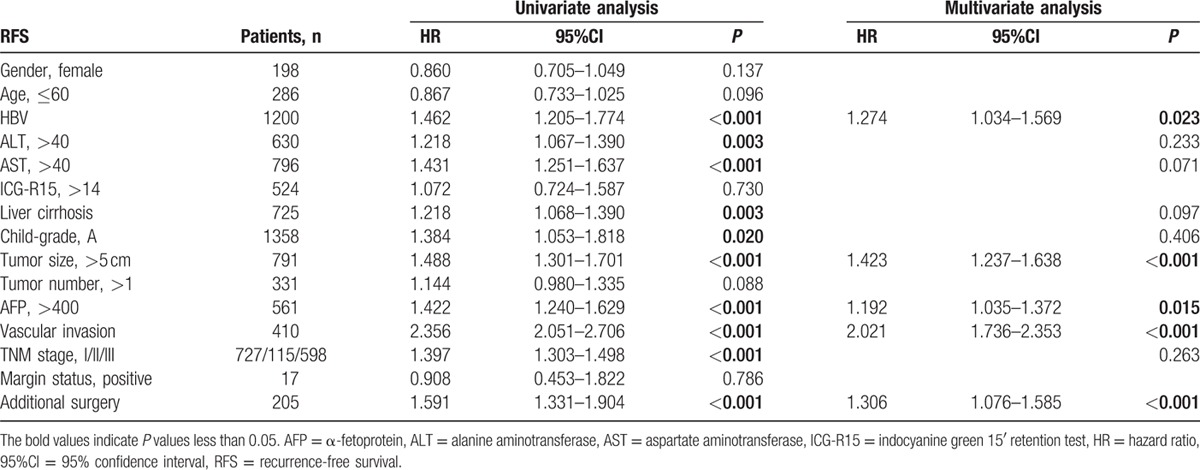
Univariate and multivariate analysis of prognostic factors for recurrence-free survival in 1440 hepatocellular carcinoma patients after hepatectomy.

**Table 4:**
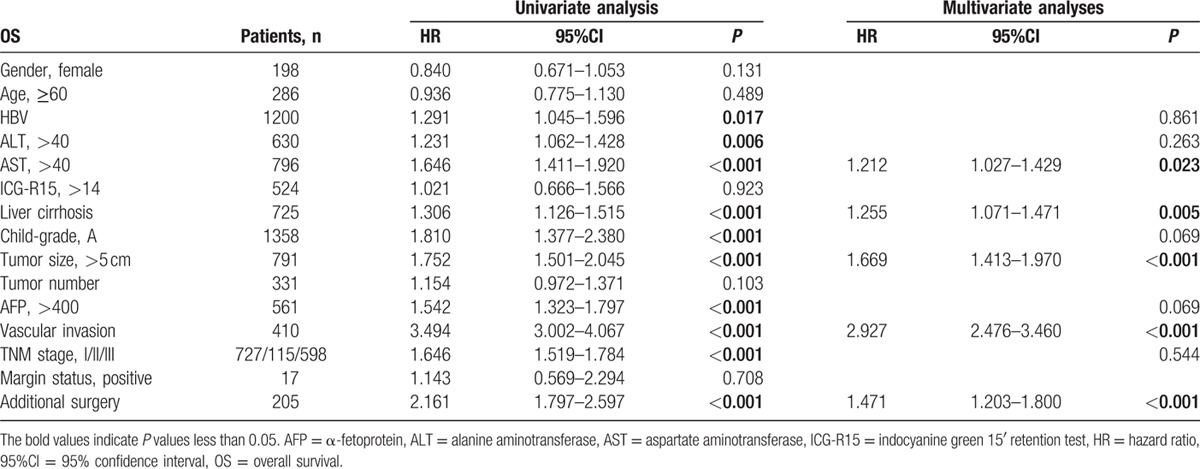
Univariate and multivariate analysis of prognostic factors for overall survival in 1440 hepatocellular carcinoma patients after hepatectomy.

### 3.5 HBV DNA levels of 10,100 and 12,800 IU/mL were the optimal cutoff points for RFS and OS in HBV-HCC patients

**Figure 3:**
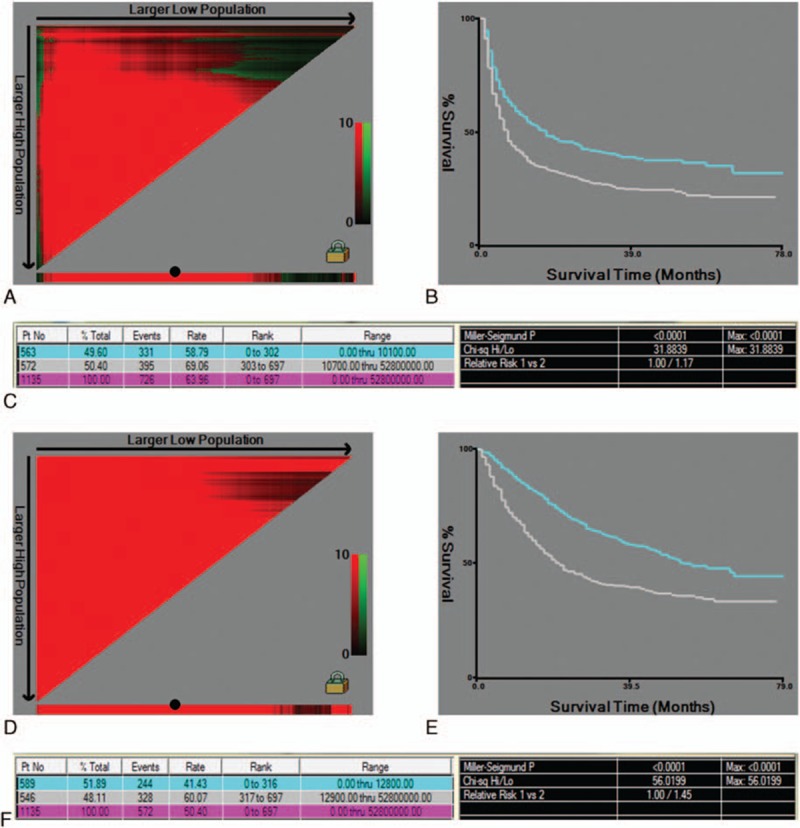
The X-tile plots for HBV DNA optimal cutoff value selection using the survival data of hepatocellular carcinoma patients with hepatitis B virus (HBV-HCC) patients. A total of 1135 HBV-HCC patients (excluding 65 HBV-HCC patients without DNA data) were enrolled in this analysis, the plot shows the χ^2^ log rank values created when the HBV DNA levels were divided into 2 groups. The optimal cutoff value, highlighted by black points on the *x*-axis (A, D), was demonstrated by Kaplan–Meier plots (B, E). Group numbers, *P* values, and other data defined by the cutoff points are shown in tables (C, F). HBV DNA was divided at the optimal cutoff value according to recurrence-free survival time (A–C) and overall survival time (D–F), with values of 10,100 and 12,800 IU/mL, respectively (*P* < 0.001).

As there is no exact method to determine an optimum HBV DNA threshold to reflect the prognostic value for the RFS and OS of HCC patients, we further assessed the association between the prognosis of HCC patients and the exact HBV DNA number using X-tile software. This software allowed us to define an optimal cutoff point that defined the exact amount of HBV DNA that predicted the prognosis of the HCC patients avoiding the arbitrary cutoff value definition. The X-tile plots provide an assessment of every division of the continuous HBV DNA data into low- and high-level expression, whereby each point on the *x*-axis represents a different cutoff point (Fig. 3A and D). The optimal HBV DNA cutoff points, as determined from the RFS and OS of the HCC set, were 10,100 and 12,800 IU/mL, respectively (Fig. 3C and F). The red color of the pixels in the X-tile plot reveals that patients with high levels of HBV DNA have a significantly worse prognosis than their counterparts, a finding that was also demonstrated in the subsequent survival curves (Fig. 3B and E).

## 4 Discussion

Different clinical features and outcomes may be reflected by different etiologies of HCC.^[23]^ We first compared the patient demographics and observed that the male was predominant in HBV-HCC patients, which may be correlated with androgen signaling in HBV and excessive alcohol consumption.^[24]^ We also found that HBV-HCC patients were significantly younger and had a significantly lower risk of comorbidities than NBC-HCC patients. A possible reason for this observation is provided by studies showing that most cases of HBV-HCC, especially in China, result from vertical transmission of HBV in infancy, thus causing HCC at a young age. In contrast, NBC-HCC develops over a long period of time.^[25,26]^

Comparisons between the 2 groups revealed that HBV-HCC patients had worse pre- and postoperative liver functions and complications. Moreover, HBV-HCC patients had more advanced TNM tumor stages. Given the close relationship between HBV and liver cirrhosis, these results are not surprising. Recently, several studies demonstrated that HBV can induce HCC.^[27,28]^ First, HBV can cause chronic cellular necrosis, inflammation, and cirrhosis, progressing to malignant transformation. Second, HBV can also directly cause HCC via interactions between a patient's genes and the DNA, X-gene product or pre-S2/S product of the HBV virus.^[29,30]^ In addition, although all of the patients had a comparable overall risk of postoperative complications, the HBV-HCC group had a significantly higher ratio of wound infections and hepatic failure (Table 2). The exact mechanisms are unknown, but poor postoperative liver function and continuous damage by the HBV virus may have contributed to this difference.

Our further survival analysis showed that HBV-HCC patients had significant lower OS and RFS rates compared with NBC-HCC patients, which is consistent with others’ previous studies,^[5,17,31]^ for example, Wang et al^[32]^ also indicated that the positive HBV-DNA patients had worse OS and RFS rates than their counterparts. The survival curves showed that HBV-HCC patients had a greater difference in their RFS rate than in their OS rate when compared with NBC-HCC patients. Interestingly, we found another more distinctive difference in the RFS curve for approximately 2 years; after that time, the HBV-HCC RFS curve decreases roughly parallel to the NBC-HCC curve. In fact, according to the time point of recurrence from the day of hepatectomy, recurrence can be classified into early-phase recurrence (within 2 years) and late-phase recurrence (more than 2 years).^[10]^ The former is mainly due to intrahepatic metastasis (IM), while the latter is mainly due to multicentric hepatocarcinogenesis (MH). IM is characterized by portal vein tumor thrombi and multiple metastases, whereas MH is a feature of novel hepatocarcinogenesis, which is mainly solitary.^[12]^ Our results also showed that the HBV-HCC group had a significantly higher ratio of early recurrence than the NBC-HCC group (Table S3), whereas the difference in their late recurrence ratio was comparable (data not shown). The fact that early recurrence occurred within 2 years makes curative treatment for early recurrence difficult while making the cure for late recurrence easier, as it can be treated by another hepatectomy, by radiofrequency ablation, or other similar treatments. Thus, early recurrence is much more deadly than late recurrence, making early recurrence the key risk factor for HCC survival, a notion that has been strongly demonstrated in the literature. These findings may be reasons why HBV-HCC patients had worse RFS and OS rates than NBC-HCC patients, an observation that was further confirmed by the comparison of stages (especially in stage II and III), we also noticed that inconsistent results were found in stage I, a stage tumor factor affect less seriously to body than stage II and III; moreover, more and more people received timely treatments (like radiofrequency ablation) because of the regular medical examinations once tumor recurrence was found, all these factors could be the possible reasons.

Many studies have demonstrated that anti-HBV therapy is beneficial in HCC patients, but none has previously clarified optimal HBV DNA levels.^[33–35]^ Despite the relationship between HBV and recurrence, the optimal preoperative HBV DNA level has remained in question, which is a very important issue in clinical practice, as the hepatitis B DNA of each HCC patient may need not be decreased to a normal level before surgery, which would waste operation time otherwise. Most previous studies have used widely varying cutoff points, such as 2 × 10^3^, 10^5^, 10^5^ to 10^6^, and 10^7^.^[5,19,36,37]^ These studies were similar in that the authors were unable to identify an optimal cutoff value using an objective and rigorous method. X-tile can determine the optimal divisions in a population by assessing every division of a continuous cohort.^[38]^ In this study, by combining the HBV DNA levels with the RFS and OS time of 1135 HBV-HCC patients (excluding 65 HBV-HCC patients without DNA data) for the first time, we showed that 1.01 × 10^4^ and 1.28 × 10^4^ were the optimal cutoff values to distinguish good or poor prognoses for recurrence and OS, respectively. Many studies have demonstrated that anti-HBV therapy would benefit the prognosis of HCC patients, but none of them clarified whether the HBV DNA should be decreased to a normal level or to a certain value.^[35,39]^ Our results have clinical implications in that HBV DNA could be decreased to approximately 10^4^ IU/mL before surgery.

Our study also has some limitations. First, although we enrolled as many patients as possible, our research is a single-center study. Second, as there were some previous studies of anti-HBV therapy and survival, we did not further test the effects of an anti-HBV therapy.^[21,22,40]^ All of the limitations above will be improved in our next study.

In conclusion, we showed that, compared with NBC-HCC patients, HBV-HCC patients had significantly worse pre- and postoperative liver function and significantly worse OS and RFS rates after hepatectomy. HBV was an independent risk factor for RFS. Finally, 10^4^ IU/mL was the optimal viral load cutoff value predicting HCC survival. Antiviral therapy should be considered before hepatectomy in patients with HBV DNA levels greater than approximately 10^4^ IU/mL.

## Supplementary Material

Supplemental Digital Content

## References

[1] Lozano R Naghavi M Foreman K Global and regional mortality from 235 causes of death for 20 age groups in 1990 and 2010: a systematic analysis for the Global Burden of Disease Study 2010. *Lancet* 2012; 380:2095–2128. 23245604 10.1016/S0140-6736(12)61728-0 PMC10790329

[2] Buendia MA Neuveut C Hepatocellular Carcinoma. *Cold Spring Harb Perspect Med* 2015; 5:1–11. 10.1101/cshperspect.a021444 PMC4315912 25646384

[3] Saitta C Tripodi G Barbera A Hepatitis B virus (HBV) DNA integration in patients with occult HBV infection and hepatocellular carcinoma. *Liver Int* 2015; 35:2311–2317. 25677098 10.1111/liv.12807

[4] Bosetti C Turati F La Vecchia C Hepatocellular carcinoma epidemiology. *Best Pract Res Clin Anaesthesiol* 2014; 28:753–770. 10.1016/j.bpg.2014.08.007 25260306

[5] Sohn W Paik YH Kim JM HBV DNA and HBsAg levels as risk predictors of early and late recurrence after curative resection of HBV-related hepatocellular carcinoma. *Ann Surg Oncol* 2014; 21:2429–2435. 24619495 10.1245/s10434-014-3621-x

[6] Bruix J Sherman M Practice Guidelines Committee AAftSoLD. Management of hepatocellular carcinoma. *Hepatology* 2005; 42:1208–1236. 16250051 10.1002/hep.20933

[7] European Association for Study of L; European Organisation for R, Treatment of C. EASL-EORTC clinical practice guidelines: management of hepatocellular carcinoma. *Eur J Cancer* 2012; 48:599–641. 22424278 10.1016/j.ejca.2011.12.021

[8] Villanueva A Llovet JM Liver cancer in 2013: mutational landscape of HCC – the end of the beginning. *Nat Rev Clin Oncol* 2014; 11:73–74. 24395088 10.1038/nrclinonc.2013.243 PMC12261303

[9] Zhou Y Sui C Li B Repeat hepatectomy for recurrent hepatocellular carcinoma: a local experience and a systematic review. *World J Surg Oncol* 2010; 8:55. 20591196 10.1186/1477-7819-8-55 PMC2904292

[10] Imamura H Matsuyama Y Tanaka E Risk factors contributing to early and late phase intrahepatic recurrence of hepatocellular carcinoma after hepatectomy. *J Hepatol* 2003; 38:200–207. 12547409 10.1016/s0168-8278(02)00360-4

[11] Wu CC Ho WL Chen JT Hepatitis viral status in patients undergoing liver resection for hepatocellular carcinoma. *Br J Surg* 1999; 86:1391–1396. 10583284 10.1046/j.1365-2168.1999.01272.x

[12] Utsunomiya T Shimada M Kudo M A comparison of the surgical outcomes among patients with HBV-positive, HCV-positive, and non-B non-C hepatocellular carcinoma: a nationwide study of 11,950 patients. *Ann Surg* 2015; 261:513–520. 25072437 10.1097/SLA.0000000000000821

[13] Kim JM Kwon CH Joh JW Outcomes after curative hepatectomy in patients with non-B non-C hepatocellular carcinoma and hepatitis B virus hepatocellular carcinoma from non-cirrhotic liver. *J Surg Oncol* 2014; 110:976–981. 25171344 10.1002/jso.23772

[14] Chan AC Chok KS Yuen WK Impact of antiviral therapy on the survival of patients after major hepatectomy for hepatitis B virus-related hepatocellular carcinoma. *Arch Surg* 2011; 146:675–681. 21690443 10.1001/archsurg.2011.125

[15] Zhou YM Zhang XF Li B Prognosis after resection of hepatitis B virus-related hepatocellular carcinoma originating from non-cirrhotic liver. *Ann Surg Oncol* 2014; 21:2406–2412. 24577811 10.1245/s10434-014-3505-0

[16] Cescon M Cucchetti A Grazi GL Role of hepatitis B virus infection in the prognosis after hepatectomy for hepatocellular carcinoma in patients with cirrhosis: a Western dual-center experience. *Arch Surg* 2009; 144:906–913. 19841357 10.1001/archsurg.2009.99

[17] Okuda Y Mizuno S Shiraishi T Clinicopathological factors affecting survival and recurrence after initial hepatectomy in non-B non-C hepatocellular carcinoma patients with comparison to hepatitis B or C virus. *Biomed Res Int* 2014; 2014:975380. 24745029 10.1155/2014/975380 PMC3972956

[18] Goto T Yoshida H Tateishi R Influence of serum HBV DNA load on recurrence of hepatocellular carcinoma after treatment with percutaneous radiofrequency ablation. *Hepatol Int* 2011; 5:767–773. 21484129 10.1007/s12072-011-9255-1

[19] Sun Y Chen TY Lu PX [Relationship between serum hepatitis B virus DNA load and hepatocellular carcinoma in Qidong, China: a cohort follow-up study of 14 years]. *Zhonghua Yi Xue Za Zhi* 2012; 92:1874–1877. 23134955

[20] Witjes CD IJzermans JN van der Eijk AA Quantitative HBV DNA and AST are strong predictors for survival after HCC detection in chronic HBV patients. *Neth J Med* 2011; 69:508–513. 22279629

[21] Yu LH Li N Shi J Does anti-HBV therapy benefit the prognosis of HBV-related hepatocellular carcinoma following hepatectomy? *Ann Surg Oncol* 2014; 21:1010–1015. 24121884 10.1245/s10434-013-3320-z

[22] Huang L Li J Yan J Antiviral therapy decreases viral reactivation in patients with hepatitis B virus-related hepatocellular carcinoma undergoing hepatectomy: a randomized controlled trial. *J Viral Hepat* 2013; 20:336–342. 23565616 10.1111/jvh.12036

[23] El-Serag HB Epidemiology of viral hepatitis and hepatocellular carcinoma. *Gastroenterology* 2012; 142:1264–1273.e1261. 22537432 10.1053/j.gastro.2011.12.061 PMC3338949

[24] De Maria N Manno M Villa E Sex hormones and liver cancer. *Mol Cell Endocrinol* 2002; 193:59–63. 12161002 10.1016/s0303-7207(02)00096-5

[25] Kaneda K Kubo S Tanaka H Features and outcome after liver resection for non-B non-C hepatocellular carcinoma. *Hepatogastroenterology* 2012; 59:1889–1892. 22819910 10.5754/hge10778

[26] Li T Qin LX Gong X Hepatitis B virus surface antigen-negative and hepatitis C virus antibody-negative hepatocellular carcinoma: clinical characteristics, outcome, and risk factors for early and late intrahepatic recurrence after resection. *Cancer* 2013; 119:126–135. 22736338 10.1002/cncr.27697

[27] Ng KY Chai S Tong M C-terminal truncated hepatitis B virus X protein promotes hepatocellular carcinogenesis through induction of cancer and stem cell-like properties. *Oncotarget* 2016; 7:24005–24017. 27006468 10.18632/oncotarget.8209 PMC5029680

[28] Tsai HW Lin YJ Wu HC Resistance of ground glass hepatocytes to oral antivirals in chronic hepatitis B patients and implication for the development of hepatocellular carcinoma. *Oncotarget* 2016; [Epub ahead of print]. 10.18632/oncotarget.8388 PMC5053683 27027237

[29] Tsai WL Chung RT Viral hepatocarcinogenesis. *Oncogene* 2010; 29:2309–2324. 20228847 10.1038/onc.2010.36 PMC3148694

[30] Schluter V Meyer M Hofschneider PH Integrated hepatitis B virus X and 3′ truncated preS/S sequences derived from human hepatomas encode functionally active transactivators. *Oncogene* 1994; 9:3335–3344. 7936659

[31] Huang G Lai EC Lau WY Posthepatectomy HBV reactivation in hepatitis B-related hepatocellular carcinoma influences postoperative survival in patients with preoperative low HBV-DNA levels. *Ann Surg* 2013; 257:490–505. 22868358 10.1097/SLA.0b013e318262b218

[32] Ming W Wei. P TianFu W Effects of hepatitis B virus load on hepatectomy. *Clin Microbiol* 2015; 4:1000205.

[33] Yang X Gao JY Wang J The impact of anti-HBV treatment on the occurrence and recurrence of hepatocellular carcinoma: focus on Asian studies. *Discov Med* 2015; 19:89–99. 25725223

[34] Honda M Shirasaki T Terashima T Hepatitis B virus (HBV) core-related antigen during nucleos(t)ide analog therapy is related to intra-hepatic HBV replication and development of hepatocellular carcinoma. *J Infect Dis* 2016; 213:1096–1106. 26621908 10.1093/infdis/jiv572

[35] Jang JW Hepatitis B virus reactivation in patients with hepatocellular carcinoma undergoing anti-cancer therapy. *World J Gastroenterol* 2014; 20:7675–7685. 24976705 10.3748/wjg.v20.i24.7675 PMC4069296

[36] Chen CF Lee WC Yang HI Changes in serum levels of HBV DNA and alanine aminotransferase determine risk for hepatocellular carcinoma. *Gastroenterology* 2011; 141:1240–1248.1248.e1241-1248.e1242. 21703214 10.1053/j.gastro.2011.06.036

[37] Nishikawa H Nishijima N Arimoto A Prognostic factors in patients with hepatitis B virus-related hepatocellular carcinoma undergoing nucleoside analog antiviral therapy. *Oncol Lett* 2013; 6:1213–1218. 24179497 10.3892/ol.2013.1578 PMC3813761

[38] Camp RL Dolled-Filhart M Rimm DL X-tile: a new bio-informatics tool for biomarker assessment and outcome-based cut-point optimization. *Clin Cancer Res* 2004; 10:7252–7259. 15534099 10.1158/1078-0432.CCR-04-0713

[39] Wei Q Xu X Ling Q Indefinite antiviral therapy may be required after surgical resection for hepatocellular carcinoma complicating chronic hepatitis B. *J Res Med Sci* 2013; 18:726–730. 24379852 PMC3872615

[40] Chong CC Wong GL Wong VW Antiviral therapy improves post-hepatectomy survival in patients with hepatitis B virus-related hepatocellular carcinoma: a prospective-retrospective study. *Aliment Pharmacol Ther* 2015; 146:675–681. 10.1111/apt.13034 25413146

