# Supplementary material for: HBV is a risk factor for poor patient prognosis after curative resection of hepatocellular carcinoma: A retrospective case–control study
Source: Medicine (Baltimore). 2016 Aug 7;95(31):e4224. doi: 10.1097/MD.0000000000004224 (PMC4979780; doi:10.1097/MD.0000000000004224)
Supplement: Supplemental Digital Content [file medi-95-e4224-s001.doc]

**Table S1** Additional surgical information in the 1440 patients with HCC who underwent hepatectomy

|  | HBV (n=1200) | NBC (n=240) | *P*-value |
| --- | --- | --- | --- |
| Biliary surgery*, n (%) | 35 (2.9%) | 33 (13.8%) | **<0.001** |
| Splenectomy, n (%) | 34 (2.8%) | 3 (1.3%) | 0.157 |
| Portal Venous Thrombectomy, n (%) | 74 (6.2%) | 10 (4.2%) | 0.228 |
| Diaphragmatic resection, n (%) | 21 (1.8%) | 3 (1.3%) | 0.782 |

The bold values indicate *P*-values less than 0.05, * Biliary surgery concludes biliary tract surgery, plastic biliary surgery and choledochojejunostomy.

**Table S2** Information of postoperative liver functions in the 1440 HCC patients after hepatectomy. ALT (A), AST (B), ALB (C), T-bil (D) and PT (E) were compared in day 1, 3, 5, 7 after surgery and the BHD.

A.

| **ALT** | Day 1 | Day 3 | Day 5 | Day 7 | BHD |
| --- | --- | --- | --- | --- | --- |
| HBV | 331(181, 600.5) | 270(147, 466) | 152(91, 275.2) | 97(60, 153) | 60(40,96) |
| NBC | 307(180, 588.5) | 233(138, 430) | 132.6(77.3, 245.8) | 82(50.5, 132.5) | 58(35, 89.5) |
| *P*-value | 0.543 | 0.084 | 0.124 | **0.007** | 0.231 |

B.

| **AST** | Day 1 | Day 3 | Day 5 | Day 7 | BHD |
| --- | --- | --- | --- | --- | --- |
| HBV | 405.5(227, 703.5) | 156(84, 300.8) | 65(45, 110) | 48(35, 70) | 46(34, 66) |
| NBC | 421(223, 680) | 117(68.3, 243.5) | 56(40, 103.8) | 42(32, 72) | 41.5(29, 62.3) |
| *P*-value | 0.895 | **0.001** | **0.024** | **0.021** | **0.006** |

C.

| **ALB** | Day 1 | Day 3 | Day 5 | Day 7 | BHD |
| --- | --- | --- | --- | --- | --- |
| HBV | 35.7(32.9, 38.3) | 34.5(31.6, 37.9) | 35.1(32.2, 38.1) | 35.3(32.1, 38.5) | 35.7(32.9, 38.3) |
| NBC | 36(33.6, 38.1) | 34.7(32.4, 34.5) | 35.3(32.4, 37.9) | 35(32.4, 37.7) | 36(33.6, 38.1) |
| *P*-value | 0.801 | 0.224 | 0.909 | 0.723 | 0.362 |

D.

| **T-bil** | Day 1 | Day 3 | Day 5 | Day 7 | BHD |
| --- | --- | --- | --- | --- | --- |
| HBV | 24.7(18, 35.1) | 24.7(18.4, 35.6) | 24.1(17.7, 36.1) | 20.2(15.2, 30.4) | 16.8(12.2, 23.1) |
| NBC | 22.1(16.4, 35.1) | 21.4(16.5, 33.1) | 21.9(16.5, 35.8) | 18.1(12.1, 30.4) | 16.9(11, 23.6) |
| *P*-value | 0.233 | **0.018** | 0.338 | 0.052 | 0.573 |

**E.**

| **PT** | Day 1 | Day 3 | Day 5 | Day 7 | BHD |
| --- | --- | --- | --- | --- | --- |
| HBV | 14.5(13.3, 16) | 14.4(13.3, 16.1) | 14.1(13, 15.7) | 14(13.1, 15.3) | 13.6(12.7, 14.9) |
| NBC | 13.8(12.7,15.1) | 13.6(12.4, 15.3) | 13.9(12.8, 14.8) | 13.5(12.8, 14.9) | 12.7(12.1, 13.7) |
| *P*-value | **0.001** | **0.000** | **0.093** | 0.215 | **0.007** |

These clinical characteristics above of the 2 groups were compared by the Mann-Whitney U test, the statistical results were showed by median (interquartile range); the bold values indicate *P*-values less than 0.05, *BHD* before hospital discharge.

**Table S3** Comparison of early recurrence in HCC patients who underwent hepatectomy

|  | HBV | NBC | *P*-value |
| --- | --- | --- | --- |
| Early recurrence |  |  | **0.001** |
| Negative | 492(41%) | 127(52.9%) |  |
| Positive | 708(59%) | 113(47.1%) |  |
